# Trichothecenes and Fumonisins: Key Players in *Fusarium*–Cereal Ecosystem Interactions

**DOI:** 10.3390/toxins16020090

**Published:** 2024-02-06

**Authors:** Alexandre Perochon, Fiona M. Doohan

**Affiliations:** UCD School of Biology and Environmental Science, UCD Earth Institute and UCD Institute of Food and Health, University College Dublin, D04 V1W8 Dublin, Ireland

**Keywords:** cereals, deoxynivalenol, Fusarium ear rot, Fusarium head blight, insects, microbiome, trichothecene

## Abstract

*Fusarium* fungi produce a diverse array of mycotoxic metabolites during the pathogenesis of cereals. Some, such as the trichothecenes and fumonisins, are phytotoxic, acting as non-proteinaceous effectors that facilitate disease development in cereals. Over the last few decades, we have gained some depth of understanding as to how trichothecenes and fumonisins interact with plant cells and how plants deploy mycotoxin detoxification and resistance strategies to defend themselves against the producer fungi. The cereal-mycotoxin interaction is part of a co-evolutionary dance between *Fusarium* and cereals, as evidenced by a trichothecene-responsive, taxonomically restricted, cereal gene competing with a fungal effector protein and enhancing tolerance to the trichothecene and resistance to DON-producing *F. graminearum*. But the binary fungal–plant interaction is part of a bigger ecosystem wherein other microbes and insects have been shown to interact with fungal mycotoxins, directly or indirectly through host plants. We are only beginning to unravel the extent to which trichothecenes, fumonisins and other mycotoxins play a role in fungal-ecosystem interactions. We now have tools to determine how, when and where mycotoxins impact and are impacted by the microbiome and microfauna. As more mycotoxins are described, research into their individual and synergistic toxicity and their interactions with the crop ecosystem will give insights into how we can holistically breed for and cultivate healthy crops.

## 1. Introduction

*Fusarium* fungi produce an array of mycotoxins that are harmful to human and animal health during the pathogenesis of some of the most economically important cereals. Among the *Fusarium* mycotoxins, trichothecenes, fumonisins and zearalenone receive a lot of attention due to both their prevalence and their toxicity. Trichothecenes are a large family of sesquiterpene epoxides that inhibit protein synthesis; those commonly found in cereal grains include deoxynivalenol (DON), DON derivatives (de-epoxy-DON, 3-acetyl-DON and 15-acetyl-DON), diacetoxyscirpenol (DAS), nivalenol (NIV), T-2 and HT-2 toxin [1]. Trichothecenes are commonly produced by the *Fusarium* species that attack cereals, with the specific metabolite produced depending on both the species and chemotype of *Fusarium* [2]. *Fusarium graminearum* (teleomorph: *Gibberella zeae*) is the most common species that attacks the floral organs of cereals, causing Fusarium ear rot (FER) on maize and Fusarium head blight (FHB) on wheat and barley. It consequently contaminates grain with DON and DON derivatives. Herein, we review recent scientific insights that enhance our understanding of the role of trichothecenes and fumonisins in fungal pathogenicity and their phytotoxic effects on plant cells. We highlight the importance of microbiome–mycotoxin and insect–mycotoxin interactions, as elucidated for trichothecenes and fumonisins, in determining the severity of plant diseases and look to future opportunities to better understand the role of *Fusarium* mycotoxins in ecosystems. 

## 2. Role of Trichothecenes and Fumonisins in Fungal Pathogenesis of Plants

Depending on the specific metabolite, host and tissue, trichothecenes stimulate the production of free radicals that cause DNA damage and interfere with many cellular processes. DON causes premature bleaching of senescing cereal heads and several studies on trichothecene-deficient *F. graminearum* mutants have elucidated that DON is a *Fusarium* virulence factor on wheat but not on maize, with conflicting results regarding its role in fungal pathogenicity on barley (reviewed in [1]). Maier et al. [3] demonstrated that, while DON is a virulence factor on wheat but not maize, NIV is a virulence factor on both crops. Hence, the authors concluded that the influence of trichothecenes on the virulence of *F. graminearum* was complex, being strongly host-specific and moderately chemotype-specific. *Fusarium* fungi also attack cereal stems and roots, and while DON plays an important role as an aggressive factor for both FHB and stem base disease, a recent study showed that trichothecene production is detrimental to wheat root colonisation by *F. graminearum* and *Fusarium culmorum* [4]. Several protein families have been shown to concurrently regulate both *Fusarium* pathogenicity and DON biosynthesis, including a Rab GTPase [5], histone acetyltransferases [6], a zinc finger transcription factor [7] and autophagy genes [8]. Mutation of the GTPase, the two histone acetyltransferases and the zinc finger transcription factor led to the down-regulation of trichothecene biosynthesis and reduced the severity of FHB, thus highlighting these genes as novel targets for controlling mycotoxin production and fungal pathogenesis.

Like DON, fumonisin B1 (FB1) is often considered to act as a necrotrophic effector, enhancing fungal colonization through its inhibition of ceramide synthesis, leading to programmed cell death (PCD) [9]. The general consensus is that FB1 is phytotoxic to susceptible genotypes of maize and plays a significant role in the pathogenicity of seedling disease; however, the B-series fumonisins do not appear to be involved in ear rot development [10]. Studies on *F. verticillioides* mutants deleted in the fumonisin gene cluster or an adjacent repressor of fumonisin production (*FvZBD1*) confirmed the role of fumonisins as a virulence factor on maize seedlings [11,12]. 

## 3. Cellular Effects of Trichothecenes and Fumonisins on Cereals

**Figure 1:**
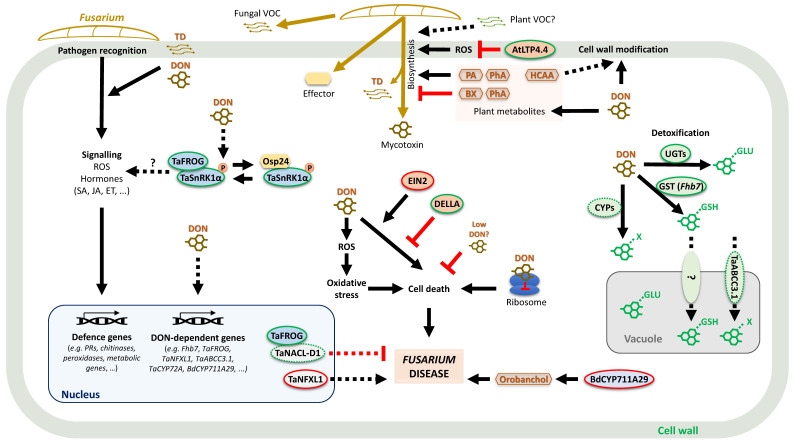
The cellular roles of mycotoxin deoxynivalenol (DON) in *Fusarium*–cereal interactions. Plant genes and pathways that are responsive to DON or influence the DON response and that are involved in *Fusarium* disease resistance and susceptibility are illustrated (reviewed by [1,14,16]). Detoxification of DON by UGTs and GST (*Fhb7*) [1,14,31] is one of the main cellular mechanisms to counteract the deleterious effects of DON, with the possible transport of modified DON into the vacuole. The signalling protein complex TaFROG/TaSnRK1α targeted by the *Fusarium* effector OSP24 is activated by DON at the transcriptional level (e.g., by *TaFROG*) or at a post-translational level (phosphorylation of TaSnRK1α) and is thought to be involved in host immunity [32,33]. DON-induced cell death is regulated by plant hormone signalling components gibberellin (DELLA) and ethylene (EIN2) [34,35]. DON induces the production of ROS and several plant defence metabolites (brown hexagons) that can influence plant cell wall composition and modulate *Fusarium* mycotoxin production [36,37,38]. It is not known yet if plant VOCs can influence DON biosynthesis. Genes are represented by oval shapes, with the inner colour depicting genes involved in detoxification (green), signalling (blue), reactive oxygen species and/or cell death (orange), or transcription factors (light grey). The green outer ring represents disease/toxin resistance genes that have been shown to enhance both DON and *Fusarium* resistance (non-dashed) or only one of the two traits (dashed). FHB susceptibility genes are illustrated with red circles. Lines represent known direct/indirect interactions (solid lines) and the hypothetical activation of downstream components (dashed lines). Black and red arrows, respectively, represent an induction or a repression of gene expression. Abbreviations: BX, benzoxazinoid; DON, deoxynivalenol; ET, ethylene; GLU, glucosyl; GSH, glutathione; HCCA, hydroxycinnamic acid amide, JA, jasmonic acid; PA, polyamine; PhA, phenolic acid; ROS, reactive oxygen species; SA, salicylic acid; TD, trichodiene; VOC, volatile organic compound.

Trichothecenes inhibit protein synthesis by interacting with the peptidyl transferase within the 60S subunit of eukaryotic ribosomes [13]. Many studies have focused on elucidating the cellular effects of DON on cereals (Figure 1). *F. graminearum* is a hemibiotroph, with a short biotrophic lifestyle phase preceding necrotrophy on plants. DON production accelerates during the switch to necrotrophic feeding, and DON secreted in advance by the invading fungus activates the production of hydrogen peroxide in plant cells; this oxidative burst activates defence responses, including phenolic acids, chitinases, glucanases and peroxidases, and the timing and location of such defence compounds influence the outcome of the fungal–plant interactions (reviewed in [14]). There is evidence that the pathogen subverts the plant metabolism to stimulate DON production: *F. graminearum* induces the synthesis of polyamines, which are themselves inducers of DON biosynthesis [15]. 

‘Omics’ research over the last two decades has identified several pathways and genes associated with DON responses in cereals, including classic defence and detoxification mechanisms [1,16] (Figure 1). A metabolomic study revealed that DON production by *F. graminearum* was necessary for full defence response activation in wheat and that the spread of a DON-deficient mutant was blocked at the rachis node, concurrent with metabolic responses that included a jasmonate-mediated defence reaction [17], which is accepted as a key hormonal system regulating the defence against hemibiotrophic pathogens [18]. The authors concluded that the delivery of DON in the rachis node may be a trigger for the switch from biotrophy to necrotrophy. Brauer et al. [19] identified some of the key transcription factors involved in the trichothecene response in wheat. This included DON-inducible TaNFXL1, which enhanced wheat susceptibility to FHB disease, as deduced using gene silencing and editing experiments. The Arabidopsis homolog of TaNFXL1, AtNFX1, was demonstrated to suppress salicylic acid- and abscisic acid-mediated defences and promote sensitivity to another trichothecene, T-2 toxin [20], and this may contribute to its effect on FHB resistance, as both hormones are linked to cereal’s defence against this disease [21].

The fumonisin FB1 disrupts sphingolipid biosynthesis and inhibits the growth of maize [9]. Maize embryo studies showed that FB1 treatment inhibited ceramide synthase, perturbing the balance of endogenous sphingolipids, disrupting membrane properties and inhibiting plasma membrane-based H^+^-ATPase activity [22]. FB1 induced ROS production, cell death and reduced jasmonic acid levels in both an FER-resistant and a FER-susceptible hybrid [23]. Iqbal et al. [9] reviewed the importance of salicylic acid, ROS and plant organelles in the plant cell response to FB1. Much of the information regarding the effects of FB1 is based on studies of the model plant *Arabidopsis thaliana*, and like trichothecenes, care must be taken in extrapolating these results to host crops. Nevertheless, these studies have shed light on the interaction between FB1 and plants. Based on Arabidopsis studies, FB1-induced cell death is positively correlated with the levels of non-phosphorylated sphingoid long-chain bases (LCBs) [24,25]. In Arabidopsis, FB1 activates a kinase cascade and free cytosolic Ca^2+^ levels, which in turn activate cellular hormones, including salicylic acid, jasmonic acid and ethylene. Two ubiquitin ligases were shown to control FB1-triggered PCD in Arabidopsis by modulating the jasmonate (JA) signalling pathway [26]. More recently, it was shown that FB1 induced a HR-like PCD event involving both oxidative and nitrosative bursts in Arabidopsis cell cultures; the up-regulated genes were those involved in the regulation of PCD, antioxidant metabolism, photosynthesis, pathogenesis and sugar transport [27]. Beyond cereals and Arabidopsis, more recent studies on fruits such as tomatoes and bananas have also increased our understanding of the cellular impacts of FB1. A study on tomatoes determined that ethylene plays an important role in the regulation of FB1-mediated cell death and defence activation, significantly affecting photosystems I and II and activating photoprotective mechanisms [28]. In bananas, FB1 biosynthesis by *F. proliferatum* contributed to fungal infection by decreasing plant defence, enhancing oxidative stress and ROS production, regulating energy metabolism and accelerating cell death [29,30].

## 4. Plant Resistance to Trichothecenes and Fumonisins 

Trichothecenes can prime host defence responses [39,40], and transcriptomic and metabolomic studies have elucidated many of the key cellular processes employed to defend against trichothecenes and reduce their phytotoxic effects [1,16] (Figure 1). The wheat quantitative trait locus (QTL) *Fhb1* on chromosome 3B is associated with enhanced DON tolerance and the accumulation of hydroxycinnamic acids [38]. The authors suggested these accumulating compounds may act as antifungal and antioxidative agents, but they are more likely used for lignification of the cell walls. Classic detoxification genes have been associated with DON and FHB resistance in wheat, including UDP-glycosyltransferase (UGTs) and glutathione transferases (GSTs) that convert DON to less toxic derivatives, cytochrome P450s (CYP450s) and multidrug resistance proteins (MRPs). Genetic approaches demonstrated this in different plants and tissues for cereal UGTs [1] and in wheat for a GST that underpins the DON and FHB resistance QTL *Fhb7* [31]. A mutation in a UGT from *Aegilops tauschii*, which is the diploid progenitor of the wheat D subgenome, affected the ability of the plant to convert DON to its less toxic derivative, DON-3-glucoside [41]. The authors speculated that the truncated version of this gene present in hexaploid wheat may increase toxin and FHB susceptibility. A wheat CYP450 gene (*TaCYP72A*) was demonstrated to enhance resistance to DON in head tissue [42] (and another was shown to be responsive to DON and DON production by *Fusarium* in wheat spikelets [43]). ABC transporters are multifunctional transmembrane proteins that use the energy from ATP hydrolysis to transport substances across the cell membrane, and wheat genes from this family (multidrug resistance and pleiotropic drug resistance proteins) have been shown to contribute to DON tolerance and FHB resistance [44,45]. Cereals also produce metabolites that suppress trichothecene production, thus reducing *Fusarium* virulence; this includes the benzoxazinoid phytoalexins (reviewed in [36]). In vitro assays demonstrated that benzoxazolinones did not directly affect growth, but they reduced DON production and the detoxification of benzoxazolinones, which is a strategy adopted by *Fusarium* to overcome wheat defences [37]. 

In addition to classic detoxification and toxin inhibition pathways, over the last decade, diverse plant genes have been shown to directly enhance DON tolerance in cereals [1,16] (Figure 1). The overexpression of a lipid transfer protein in wheat inhibited both the spread of *F. graminearum* and DON accumulation in wheat spikes and significantly increased the resistance of transgenic wheat leaves to DON-induced oxidative stress [46]. A DON-activated wheat SnRK1 (TaSnRK1 a) and a *Poaceae*-divergent NAC transcription factor enhanced FHB resistance when overexpressed or silenced in wheat [47,48]. Both of these interact with a novel DON-inducible *Pooideae*-specific protein, TaFROG, which itself also enhances DON tolerance and FHB resistance when overexpressed in wheat [33]. TaFROG competes with a fungal orphan protein effector, Osp24, to stabilise the central stress regulator TaSnRK1a [32]. This was the first report of competing plant and fungal orphan proteins playing a key role in plant–pathogen interactions and highlights the fact that mycotoxins play an important role in mediating the co-evolution of cereals and *Fusarium* fungi. 

Many other genes are postulated to enhance DON resistance and/or interact with DON to enhance FHB resistance, and the validation of their effects would greatly enhance the repertoire of genes available to cereal breeders who work to pyramid disease resistance into varieties. For example, Sun et al. [49] recently identified a *Fusarium*-responsive plant laccase (TaLAC78). Laccases catalyse the oxidative polymerization of monolignols, reinforcing cell walls, and they postulated that TaLAC78 may enhance wheat resistance to *F. graminearum*. Molecular docking studies demonstrated that TaLAC78 may possibly interact with DON in addition to playing a role in lignin biosynthesis. More gene families have been shown to enhance DON tolerance in other organisms, and while they exist in cereals, their role in FHB resistance has not been demonstrated. For example, aldo-keto reductases (AKR) have been shown to degrade DON in microbes [50]. While there is no evidence to date that AKRs degrade DON in wheat, genes encoding AKRs were linked with a FHB resistance QTL on chromosome 3B of wheat [51]. 

Ethylene signalling is an important factor in sphingolipid synthesis, and it has been shown to partially rescue FB1-induced cell death in tomatoes in Arabidopsis [28,52]. Relatively few studies have elucidated maize mechanisms that confer tolerance to fumonisins. While many QTL for reduced fumonisin content in maize have been identified [53], no studies have yet linked any of these to enhanced fumonisin tolerance or degradation. Only one gene associated with FER resistance has been cloned, *ZmAuxRP1*, which underpins the quantitative disease-resistance locus *qRfg2* in maize, and it encodes an auxin-regulated protein that may indirectly regulate fumonisin production by modulating indole-3-glycerol phosphate and/or the indole flux at the branch point between the IAA and benzoxazinoid biosynthetic pathways [54]. Studies on other plants have given insights into potential FB1 resistance pathways in plants (reviewed in [55]). Serine palmitoyltransferase (SPT) mediates FB1-initiated PCD by catalysing sphingolipid biosynthesis; SPT functions as a heterodimer, and a dominant mutant allele has been characterised that confers FB1 resistance in Arabidopsis [56]. Also, the SBT-interacting protein (*ssSPTa)* stimulates sphingolipid synthesis, and the overexpression of this protein reduced tolerance to FB1, while *ssSPTa* RNA interference lines displayed enhanced tolerance to FB1 [57]. Increased salicylic acid production in maize in response to FB1 treatment was observed in a FER-resistant hybrid but not in an FER-susceptible hybrid [23], and hence it is likely that this hormonal pathway and its associated genes play a role in defence against FB1.

## 5. *Fusarium* Mycotoxin–Microbiome and Mycotoxin–Insect Interactions

**Figure 2:**
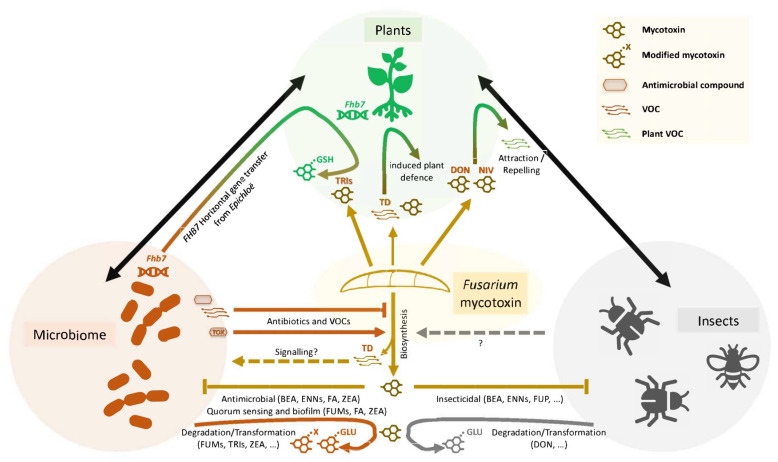
Interplay between *Fusarium* mycotoxins and the plant ecosystem. Plant and *Fusarium* interactions with microbiomes (soil- and plant-associated microorganisms), as well as insects, impact mycotoxin production and modify mycotoxins. Bacteria and fungi produce antibiotics and unidentified VOCs that inhibit mycotoxin biosynthesis [58]. Volatile TD is proposed to negatively regulate trichothecene biosynthesis and act as an intra/interspecies signalling molecule affecting microbial pathogen virulence and plant defence [39]. Conversely, the antimicrobial toxin toxoflavin produced by the plant bacterial pathogen *Burkholderia glumae* promotes DON production [64]. As reviewed by Venkatesh and Keller [58], mycotoxins alter the microbiome via their antimicrobial activities and by modulating quorum sensing and biofilm formation, and in return, microbes can degrade and transform mycotoxins via oxidation, epimerisation, de-epoxidation and glycosylation. Mycotoxins also have insecticidal activities [58], and aphids can convert DON to the less toxic DON-3-glucoside [67]. Mycotoxins indirectly augment insect behaviour by manipulating the production of VOCs that attract or repel insects [71,75]. It remains to be determined if insects manipulate mycotoxin biosynthesis. Close physical interactions between plants and microbes may have facilitated the transfer of the FHB resistance gene *FHB7* from endophytic fungi to grasses [31], offering plants a new gene to detoxify trichothecenes. Lines represent positive effects or activities that are known (solid line) or hypothesised (dashed). Mycotoxins associated with each activity are given in parenthesis. Abbreviations: BEA, beauvericin; DON, deoxynivalenol; ENNs, enniatins; FUMs, fumonisins; FA, fusaric acid; FUP, fusaproliferin; GLU, glucosyl; GSH, glutathione; NIV, nivalenol; TD, trichodiene; TOX, toxoflavin; TRIs, trichothecenes; VOC, volatile organic compound; ZEA, zearalenone.

**Figure 3:**
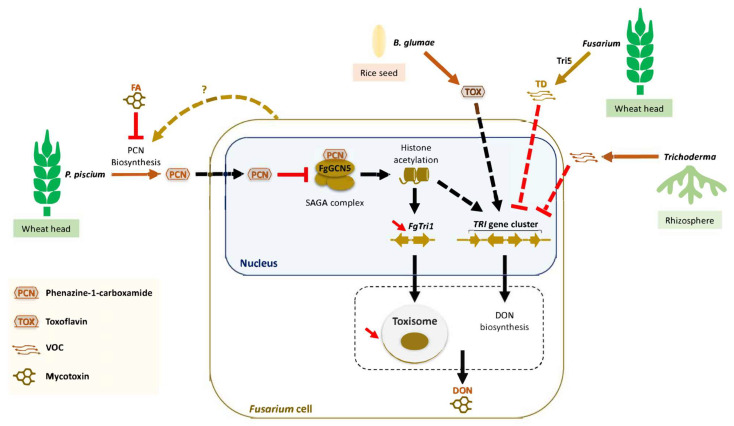
Mode of action of bioactive microbial compounds that modulate *Fusarium* mycotoxin production. Plant microbiomes contain beneficial or pathogenic microorganisms that produce compounds to modulate mycotoxin production by *Fusarium*. The wheat head microbiome bacterium *Pseudomonas piscium* (strain ZJU60) interacts with *Fusarium graminearum* [61]. *P. piscium* secretes phenazine-1-carboxamide (PCN) that enters *Fusarium* cells and inhibits the histone acetyltransferase activity of FgGcn5, likely through direct binding. Subsequently, histone acetylation is deregulated, leading to transcriptional suppression of the trichothecene biosynthesis gene *FgTri1*, disruption of the DON biosynthesis toxisome and reduced production of DON [61]. It is possible that other genes in the trichothecene biosynthetic pathway were also deregulated, as these genes are activated by Gcn5-dependent histone acetylation under DON-inducing growth conditions [6]. When *P. piscium* is co-cultivated with *Fusarium*, PCN production is increased, suggesting that this fungal–bacterial interaction stimulates PCN production [61]. Interestingly, PCN biosynthesis by *Pseudomonas* species has been reported to be repressed by *Fusarium* mycotoxin fusaric acid in another *Pseudomonas* (*P. chlororaphis*) [60]. *F. graminearum* and the seed-borne bacterial pathogen *Burkholderia glumae* interact cooperatively to promote their dispersal and disease progression on rice plants [64]. Toxoflavin is produced by *B. glumae* during this interaction, and it increases the expression of genes in the trichothecene biosynthetic pathway and DON production. Recently, fungal VOCs have been shown to inhibit the expression of trichothecene biosynthesis genes and mycotoxin production [39,63], but their identities and their exact molecular effects remain to be determined. Lines represent known direct/indirect positive effect or positive activation (solid lines) and hypothetical activation of downstream components (dashed lines). Red downward arrows represent an activity decrease. Abbreviations: DON, deoxynivalenol; FA, fusaric acid; FUM, fumonisin; PCN, phenazine-1-carboxamide; SAGA, Spt-Ada-Gcn5 acetyltransferase; TD, trichodiene; TOX, toxoflavin; TRI, trichothecene; VOC, volatile organic compound.

As recently reviewed by Venkatesh and Keller [58], there is growing evidence that a diverse array of *Fusarium* mycotoxins possess antibacterial and antifungal activities that help these fungi combat other plant microbes. Some mycotoxins have more subtle ecological functions, such as modulating quorum sensing and biofilm formation. Figure 2 illustrates the importance of mycotoxins as determinants of the interactions between microbes, plants and insects. DON and other *Fusarium* mycotoxins have been shown to repress pathways that contribute to the biocontrol activity of fungi and bacteria [39,59,60]. Trichodiene is a volatile metabolite produced in the first step of the trichothecene biosynthetic pathway; when it was expressed in the biocontrol fungus *Trichoderma*, it negatively regulated trichothecene biosynthesis by *F. graminearum*, inhibiting the expression of trichothecene biosynthetic genes and DON accumulation [39] (Figure 3). Aside from its role in trichothecene biosynthesis, it was proposed that trichodiene can act as an intra- and interspecies signal to modulate pathogen virulence and host plant resistance [39]. More research is needed to confirm this role and elucidate all the biological roles of trichodiene.

*Fusarium* mycotoxins can negatively or positively impact cohabiting microbes. Microbes have evolved strategies to manipulate the production or bioactivity of toxins, as overviewed in Figure 3. The bacterium *Pseudomonas piscium* is a member of the wheat head microbiome, and it secretes phenazine-1-carboxamide, which inhibits the histone acetyltransferase activity of a component of Spt-Ada-Gcn5 acetyltransferase (SAGA), consequently suppressing growth, pathogenicity and DON biosynthesis in *F. graminearum* [61]. The maize seed endophyte *Sarocladium zeae* produces pyrrocidines, which inhibited the growth of *F. verticillioides* and induced the expression of the fumonisin repressor *FvZBD1* [11]. Hence, *S. zeae* may influence fumonisin production to influence competitor pathogenicity. Volatile organic compounds (VOCs) emitted by soil- and plant-associated microorganisms influence the production of fungal toxins, including DON and zearalenone, by *Fusarium* fungi. As mentioned above, the trichothecene trichodiene inhibits DON biosynthesis (Figure 3), but other volatiles produced by antagonistic fungi also inhibit trichothecene production. Diverse endophytic and antagonistic fungi produced volatiles that suppressed DON, 15-acetyl DON and zearalenone production, although the effects on DON and 15-acetyl DON were species-specific [62]. *Trichoderma* VOCs suppressed type A trichothecenes, fumonisins and fusaric acid production by *F. sporotrichioides* and *F. verticillioides*, and this is likely due to the inhibition of mycotoxin biosynthesis (rather than biotransformation) because VOCs inhibit the expression of trichothecene and fumonisin biosynthetic genes [63]. This is an emerging area of research, and there is potential to identify novel VOCs that can function as disease-control agents through the suppression of toxin biosynthesis during FHB and FER. One of the most intriguing findings in recent years was that DON is an important component of cooperative interactions between pathogens. *F. graminearum* cooperates with another rice pathogen, *Burkholderia glumae*, each facilitating the others’ disease progression [64]. Disease severity and *F. graminearum* DON production were increased on rice heads when co-inoculated with *B. glumae*. This may be in part due to the ability of the bacterial virulence factor toxoflavin to increase trichothecene gene expression, DON production and spore production by *F. graminearum.* The authors hypothesised that this would in turn help in the aerial dispersal of *B. glumae* since its cells were found to be physically attached to *F. graminearum* spores. Additionally, they proposed that toxoflavin’s antimicrobial activity may suppress competitors of *Fusarium* fungi. 

Soil- and plant-associated microorganisms can degrade and/or transform *Fusarium* mycotoxins, including fumonisins, zearalenone and trichothecenes, into less toxic forms [65]. There is some evidence that microbes, plants and insects use similar self-protection mechanisms against mycotoxins (Figure 2). Like plants, endophytic *Trichoderma* and aphids glycosylate DON to the less toxic DON-3-glucoside [66,67]. *Trichoderma* has the capacity to glycosylate various trichothecenes and transform zearalenone into less toxic sulphated forms [63,68]. As mentioned above, the QTL *Fhb7* encodes a glucosyltransferase, and this was introgressed into wheat from an endophytic *Epichloë* species via horizontal gene transfer (HGT) [31]. This strongly suggests that *Epichloë* and plant species use similar mechanisms to detoxify trichothecenes. These findings suggest that HGT could play an important role in disarming fungal toxins, and a similar phenomenon occurred in insect–plant interactions wherein a plant detoxification gene was hijacked by the whitefly pest to neutralise plant toxins [69].

*Fusarium* fungi interact with insects as pathogens [70] and use them as a vector for disease spread [71]. But, as reviewed by Drakulic et al. [71], there are many other aspects to *Fusarium*–insect interactions, some of which influence mycotoxin levels and some of which are mediated by mycotoxins (Figure 2). Insects and mites that interact with *Fusarium* fungi in infected wheat grain show variable tolerance levels to *Fusarium* fungi (which differ in their mycotoxin profiles) [72]. The role of mycotoxins or mycotoxin-induced host biochemicals (e.g., VOCs) in the insect/mite–*Fusarium* relationship warrants further investigation. Several studies investigated the role of trichothecenes in the interactions between the aphid *Sitobion avenae*, *Fusarium* and wheat (reviewed in Drakulic et al. [71]). Aphids were shown to enhance disease severity and trichothecene production by *F. graminearum* and *Fusarium langsethiae* on wheat [73,74]. *F. graminearum*-infected wheat ears emitted VOCs that were repellent to aphids, whereas *F. langsethiae* infection did not change the insect behaviour [73,74]. Wheat infected with a DON-producing *F. graminearum* isolate was repellent to aphids, whereas infection with a NIV-producing isolate attracted them. Further studies on the relationship between mycotoxin-induced host VOCs and insects will help unravel the complexity of mycotoxin–host–insect interactions.

## 6. Conclusions and Outlook

Over the last few decades, we have greatly advanced our understanding of plant–mycotoxin interactions based on binary mycotoxin–plant studies. While there is compelling evidence that mycotoxins have multiple effects on plant cells, one of the major unknowns is how plant cells uptake many mycotoxins. For nearly 20 years, it has been known that trichothecenes are produced and diffused in the host tissues in advance by the invading fungal hyphae [76]. But it is unknown whether their uptake into plant cells is passive, active or both. Another area that warrants further investigation is the synergic phytotoxic effects of mycotoxins. As shown by Wipfler et al. [77], culmorin and trichothecene mycotoxins have synergistic phytotoxic effects, and both often co-occur in cereals, both being produced by many of the same *Fusarium* fungi. Recent insights into mycotoxin–plant–microbiome and mycotoxin–plant–insect interactions suggest that we need more ecosystem-based holistic studies to refine our understanding of the role of mycotoxins and to optimise mycotoxin mitigation strategies. Specifically, we need to (i) better define the ecological roles of mycotoxins and the antagonistic and synergistic relationships between toxin and microbes/insects, (ii) understand the potential synergistic effects of mycotoxins on plant cells and (iii) understand the role of VOCs in mycotoxin–ecosystem interactions. Recent advances in omics technologies mean we now have the tools to answer these questions. From a host perspective, we have the tools to determine whether we can breed for a healthy microbiome/ecosystem that protects host plants from the deleterious effects of mycotoxins.

## Data Availability

No new data were created or analyzed in this study. Data sharing is not applicable to this paper.
